# Cannabis-Involved Emergency Department Visits Among Persons Aged <25 Years Before and During the COVID-19 Pandemic — United States, 2019–2022

**DOI:** 10.15585/mmwr.mm7228a1

**Published:** 2023-07-14

**Authors:** Douglas R. Roehler, Herschel Smith, Lakshmi Radhakrishnan, Kristin M. Holland, Abigail L. Gates, Alana M. Vivolo-Kantor, Brooke E. Hoots

**Affiliations:** ^1^Division of Overdose Prevention, National Center for Injury Prevention and Control, CDC; ^2^Office of Public Health Data, Surveillance, and Technology, CDC; ^3^Division of Violence Prevention, National Center for Injury Prevention and Control, CDC.

**Summary**

**What is already known about this topic?**

Cannabis-involved emergency department (ED) visits increased for youths aged 0–14 years before 2019, as cannabis legalization expanded across the United States.

**What is added by this report?**

Cannabis-involved ED visits among young persons were higher during the COVID-19 pandemic than during 2019. Large increases in cannabis-involved ED visit rates occurred among children aged ≤10 years, and among persons aged 11–14 years; rates among females aged 11–14 years increased more than they did among males.

**What are the implications for public health practice?**

To protect youths from unintentional ingestions, it is important that safe cannabis storage practices be employed in households. Local implementation of youth- and young adult–focused evidence-based programs to improve coping with stressors might prevent initiation and continued use of cannabis, and modifications in cannabis packaging might decrease its appeal to youth, as cannabis use policies continue to increase cannabis availability in some states.

## Abstract

To understand trends in U.S. cannabis-involved emergency department (ED) visits (i.e., those for which cannabis use was documented in the chief complaint or a discharge diagnosis) among young persons aged <25 years during the COVID-19 pandemic, CDC used National Syndromic Surveillance Program data to examine changes in ED visits during 2019–2022. Mean weekly cannabis-involved ED visits among all young persons were higher during the COVID-19 pandemic in 2020, 2021, and 2022, compared with corresponding periods in 2019. Large increases in cannabis-involved ED visits throughout the COVID-19 pandemic compared with prepandemic surveillance periods in 2019 were identified among persons aged ≤10 years. ED visit rates among children and adolescents aged 11–14 years did not differ by sex until the first half of the 2020–21 school year (2020, weeks 37–53), when ED visit rates among females surpassed those among males. Improving clinicians’ awareness of rising cannabis-involved ED visits might aid in early diagnosis of cannabis intoxication among young persons. Further, increasing adults’ knowledge regarding safe cannabis storage practices, strengthening youths’ coping and problem-solving skills through evidence-based prevention programs, and modifying cannabis packaging to decrease appeal to youths might help prevent intentional and unintentional cannabis use.

## Introduction

Approximately 18.7% of U.S. persons aged ≥12 years used cannabis in 2021.* Expansion of legalization of medical and nonmedical cannabis^†^ has contributed to increased availability and use of cannabis by adults (*1*), and Monitoring the Future data show that youth’s perception of the risk of cannabis use has declined.^§^ The COVID-19 pandemic has been associated with increases in substance use for some youths (*2*); however, cannabis-involved emergency department (ED) visits began increasing statistically significantly several years before the start of the pandemic among all age groups except ages 15–24 years (*3*).

## Methods

CDC analyzed data from a weekly average of 1,671 EDs consistently reporting data^¶^ to the National Syndromic Surveillance Program (NSSP).**^,^^††^ In collaboration with state and local health departments, CDC developed and validated a definition for cannabis-involved ED visits using *International Classification of Diseases, Tenth Revision, Clinical Modification* diagnosis codes F12.1, F12.2, F12.9, or T40.7; or chief complaint text indicating cannabis use (e.g., “smoke weed” or “ingest hash”) (Supplementary Box, https://stacks.cdc.gov/view/cdc/130568). Changes in cannabis-involved ED visit rates among persons aged <25 years between 2019 and 2022 were quantified and stratified by age group and sex, using four metrics: 1) mean weekly number of cannabis-involved ED visits, 2) rates of cannabis-involved ED visits (number of cannabis-involved ED visits per 10,000 ED visits),^§§^ 3) overall visit ratios^¶¶^ and for each sex by year (calculated as the rate of ED visits that were cannabis-involved during the study period divided by the rate during the 2019 reference period), and 4) visit ratios by sex*** (calculated as the rate of female ED visits that were cannabis-involved divided by the rate of male ED visits within the same period). Four periods during 2020, 2021, and 2022 were analyzed: weeks 1–11 (prepandemic in 2020),^†††^ weeks 12–23 (second half of school year), weeks 24–36 (summer), and weeks 37–53 (first half of school year). Overall visit ratios were calculated comparing ED visit rates during these periods in 2020-2022 with rates for the corresponding epidemiologic weeks in 2019. Analyses were conducted using R software (version 4.2.2; R Foundation). This activity was reviewed by CDC and was conducted consistent with applicable federal law and CDC policy.^§§§^

## Results

During December 30, 2018–January 1, 2023, a total of 539,106 cannabis-involved ED visits occurred among persons aged <25 years (64.9 per 10,000 ED visits) in the United States. During the pandemic, the average number of weekly cannabis-involved ED visits involving persons aged ≤10 years ranged from 30.4 (2020, weeks 12–23) to 71.5 (2022, weeks 24–36), compared with the prepandemic periods (range = 18.7 [2019, weeks 1–11] to 23.2 [2020, weeks 1–11]) (Table 1). Among persons aged 11–14 years, the mean number of weekly cannabis-involved ED visits during the pandemic ranged from 69.8 (2020, weeks 12–23) to 209.3 (2022, weeks 12–23), compared with 90.5 (2019, weeks 24–36) to 138.5 (2020, weeks 1–11) during the prepandemic period. Among adolescents and young adults aged 15–24 years, the average weekly number of cannabis-involved ED visits during the pandemic ranged from 2,275.8 (2020, weeks 12–23) to 2,813.2 (2021, weeks 12–23), compared with 2,117.5 (2019, weeks 1–11) to 2,531.1 (2020, weeks 1–11) during the prepandemic period.

**TABLE 1:** Average weekly number of cannabis-involved* emergency department visits^†^ among persons aged <25 years, by age group and sex — National Syndromic Surveillance Program, United States, 2019–2022

Year and epidemiologic weeks^§^	Average weekly no. of cannabis-involved ED visits by age group, yrs								
	≤10			11–14			15–24		
	All	Females	Males	All	Females	Males	All	Females	Males
**2019**									
1–11	**18.7**	9.5	9.3	**105.1**	49.9	55.1	**2,117.5**	914.9	1,198.1
12–23	**21.9**	11.2	10.8	**113.9**	51.7	62.1	**2,316.1**	1,007.8	1,303.1
24–36	**20.8**	10.3	10.5	**90.5**	41.9	48.2	**2,223.5**	977.6	1,240.3
37–53	**22.2**	10.7	11.5	**120.6**	58.3	62.1	**2,426.1**	1,053.6	1,366.3
**2020**									
1–11 (prepandemic)	**23.3**	10.8	12.4	**138.5**	67.8	70.5	**2,531.1**	1,119.5	1,403.7
12–23 (second half of school year)	**30.4**	14.3	16.0	**69.8**	37.3	32.4	**2,275.8**	1,013.2	1,257.4
24–36 (summer)	**42.8**	20.9	21.8	**90.8**	47.0	43.6	**2,555.7**	1,177.3	1,370.8
37–53 (first half of school year)	**40.3**	20.1	20.3	**96.4**	52.7	43.2	**2,364.0**	1,095.6	1,261.7
**2021**									
1–11	**48.5**	24.9	23.5	**108.4**	63.1	44.9	**2,533.1**	1,205.8	1,319.5
12–23 (second half of school year)	**67.0**	32.6	34.3	**133.3**	76.8	55.8	**2,813.2**	1,347.2	1,456.1
24–36 (summer)	**63.3**	30.5	32.7	**97.0**	56.8	40.0	**2,373.5**	1,140.2	1,225.6
37–53 (first half of school year)	**47.4**	24.1	23.3	**147.3**	88.0	58.9	**2,309.2**	1,128.4	1,173.3
**2022**									
1–11	**57.9**	27.4	30.5	**184.5**	112.9	71.0	**2,351.1**	1,131.0	1,211.7
12–23 (second half of school year)	**66.7**	30.7	35.8	**209.3**	126.0	83.3	**2,774.3**	1,385.8	1,380.4
24–36 (summer)	**71.5**	36.2	35.1	**127.0**	75.0	51.6	**2,345.9**	1,157.8	1,174.6
37–53 (first half of school year)	**66.1**	33.6	32.2	**187.8**	110.9	76.5	**2,314.5**	1,147.7	1,156.1

**Abbreviations:** ED = emergency department; NSSP = National Syndromic Surveillance Program.

* NSSP collects free-text reason for visit (chief complaint), discharge diagnosis, and patient demographic details. Free-text keywords and diagnostic codes combined using Boolean searches were used to create a keyword syndrome to identify ED visits involving cannabis. CDC developed and validated a definition for cannabis-involved ED visits using *International Classification of Diseases, Tenth Revision, Clinical Modification* diagnosis codes F12.1, F12.2, F12.9, or T40.7 or chief complaint text indicating cannabis use (e.g., “smoke weed” or “ingest hash”).

^†^ NSSP receives anonymized medical record information from approximately 75% of nonfederal EDs nationwide. To reduce the artifactual impact of changes in reporting patterns, analyses were restricted to facilities with more consistent reporting of more complete data (coefficient of variation ≤40 and average weekly informative discharge diagnosis ≥75% complete during 2019–2022).

^§^ A standardized method of counting weeks to allow for the comparison of data year after year; epidemiologic weeks start on a Sunday and end on a Saturday. Four periods were analyzed: prepandemic (epidemiologic weeks 1–11), second half of school year (epidemiologic weeks 12–23), summer (epidemiologic weeks 24–36), and first half of school year (epidemiologic weeks 37–53).

Among children aged <10 years, the pandemic peak in mean weekly visits (71.5) occurred during the summer of 2022 (weeks 24–36). During the pandemic, cannabis-involved ED visit rates among children aged ≤10 years began declining during the second half of the 2020–21 school year (2021, weeks 12–23), but increased thereafter, peaking during the summer of 2022 (weeks 24–36) at 4.0. Cannabis-involved ED visit ratios per 10,000 ED visits in this age group ranged from 2.4 (2021, weeks 37–53) to 5.8 (2021, weeks 1–11) (Table 2).

**TABLE 2:** Average weekly rate* and visit ratio^†^ of cannabis-involved emergency department visits^§^ among persons aged <25 years, by age group — National Syndromic Surveillance Program, United States, 2019–2022

Period, epidemiologic weeks^¶^ and metric	Age group, yrs											
	≤10				11–14				15–24			
	All	Females	Males	Visit ratio (95% CI)** by sex^††^	All	Females	Males	Visit ratio (95% CI)** by sex**^††^**	All	Females	Males	Visit ratio (95% CI)** by sex^††^
**Cannabis-involved ED visit rate**												
1–11, 2019	**0.9**	0.9	0.8	1.2 (0.9–1.5)	**21.2**	19.5	23.0	0.8 (0.8–1.0)	**106.5**	73.8	160.7	0.5 (0.4–0.5)
12–23, 2019	**1.1**	1.2	1.0	1.2 (1.0–1.5)	**22.5**	20.4	24.6	0.8 (0.7–0.9)	**112.9**	80.1	165.2	0.5 (0.5–0.5)
24–36, 2019	**1.2**	1.3	1.1	1.2 (0.9–1.5)	**21.9**	20.7	23.0	0.9 (0.8–1.0)	**109.4**	79.1	156.2	0.5 (0.5–0.5)
37–53, 2019	**1.0**	1.1	1.0	1.1 (0.9–1.3)	**23.2**	22.9	23.5	1.0 (0.9–1.1)	**116.4**	82.3	170.8	0.5 (0.5–0.5)
1–11, 2020	**1.0**	1.0	1.0	1.0 (0.8–1.3)	**25.8**	24.6	27.1	0.9 (0.8–1.0)	**119.5**	85.8	173.3	0.5 (0.5–0.5)
12–23, 2020	**4.2**	4.3	4.1	1.1 (0.9–1.3)	**38.9**	40.5	37.3	1.1 (0.9–1.2)	**188.2**	142.2	254.0	0.6 (0.5–0.6)
24–36, 2020	**4.5**	4.8	4.3	1.1 (1.0–1.3)	**32.4**	32.8	31.9	1.0 (0.9–1.2)	**151.4**	116.7	202.6	0.6 (0.6–0.6)
37–53, 2020	**4.1**	4.5	3.8	1.2 (1.0–1.4)	**32.4**	34.2	30.3	1.1 (1.0–1.3)	**143.5**	108.8	197.7	0.6 (0.5–0.6)
1–11, 2021	**5.0**	5.5	4.5	1.2 (1.0–1.5)	**37.7**	40.6	34.0	1.2 (1.1–1.3)	**162.0**	125.4	220.1	0.6 (0.6–0.6)
12–23, 2021	**4.6**	4.9	4.3	1.1 (1.0–1.3)	**34.9**	39.1	30.0	1.3 (1.2–1.4)	**152.9**	120.9	201.7	0.6 (0.6–0.6)
24–36, 2021	**3.6**	3.7	3.4	1.1 (1.0–1.3)	**23.9**	27.8	19.8	1.4 (1.3–1.6)	**117.7**	93.6	154.3	0.6 (0.6–0.6)
37–53, 2021	**2.5**	2.8	2.2	1.2 (1.1–1.4)	**32.2**	38.3	26.0	1.5 (1.4–1.6)	**117.7**	94.5	153.8	0.6 (0.6–0.6)
1–11, 2022	**3.8**	3.9	3.7	1.0 (0.9–1.2)	**45.5**	53.2	36.9	1.4 (1.3–1.6)	**133.6**	105.1	178.2	0.6 (0.6–0.6)
12–23, 2022	**3.4**	3.4	3.4	1.0 (0.9–1.2)	**44.4**	53.3	35.5	1.5 (1.4–1.6)	**144.6**	119.0	184.1	0.6 (0.6–0.7)
24–36, 2022	**4.0**	4.5	3.6	1.2 (1.1–1.4)	**32.5**	39.3	25.9	1.5 (1.4–1.7)	**122.4**	100.2	154.4	0.7 (0.6–0.7)
37–53, 2022	**2.7**	2.9	2.4	1.2 (1.1–1.4)	**35.8**	43.2	28.7	1.5 (1.4–1.6)	**117.6**	96.1	150.1	0.6 (0.6–0.7)
Cannabis-involved ED visit ratio (95% CI)** during pandemic compared to corresponding surveillance periods in 2019												
1–11, 2020^§§^	**1.0**	1.0	1.0	—	**25.8**	24.6	27.1	—	**119.5**	85.8	173.3	—
12–23, 2020	**4.0 (3.4–4.6)**	3.7 (2.9–4.6)	4.2 (3.4–5.3)	—	**1.7 (1.6–1.9)**	2.0 (1.8–2.2)	1.5 (1.3–1.7)	—	**1.7 (1.6–1.7)**	1.8 (1.7–1.8)	1.5 (1.5–1.6)	—
24–36, 2020	**3.7 (3.2–4.3)**	3.6 (3.0–4.5)	3.8 (3.1–4.6)	—	**1.5 (1.4–1.6)**	1.6 (1.4–1.8)	1.4 (1.2–1.6)	—	**1.4 (1.4–1.4)**	1.5 (1.4–1.5)	1.3 (1.3–1.3)	—
37–53, 2020	**4.1 (3.6–4.7)**	4.3 (3.6–5.2)	4.0 (3.3–4.8)	—	**1.4 (1.3–1.5)**	1.5 (1.4–1.6)	1.3 (1.2–1.4)	—	**1.2 (1.2–1.3)**	1.3 (1.3–1.4)	1.2 (1.1–1.2)	—
1–11, 2021	**5.8 (5.0–6.9)**	6.0 (4.8–7.5)	5.6 (4.5–7.1)	—	**1.8 (1.6–1.9)**	2.1 (1.9–2.3)	1.5 (1.3–1.7)	—	**1.5 (1.5–1.6)**	1.7 (1.7–1.7)	1.4 (1.3–1.4)	—
12–23, 2021	**4.4 (3.8–5.0)**	4.2 (3.5–5.1)	4.5 (3.7–5.5)	—	**1.5 (1.4–1.7)**	1.9 (1.7–2.1)	1.2 (1.1–1.4)	—	**1.4 (1.3–1.4)**	1.5 (1.5–1.6)	1.2 (1.2–1.3)	—
24–36, 2021	**2.9 (2.5–3.3)**	2.8 (2.3–3.4)	3.0 (2.5–3.6)	—	**1.1 (1.0–1.2)**	1.3 (1.2–1.5)	0.9 (0.8–1.0)	—	**1.1 (1.1–1.1)**	1.2 (1.2–1.2)	1.0 (1.0–1.0)	—
37–53, 2021	**2.4 (2.2–2.8)**	2.6 (2.2–3.1)	2.3 (2.0–2.8)	—	**1.4 (1.3–1.5)**	1.7 (1.5–1.8)	1.1 (1.0–1.2)	—	**1.0 (1.0–1.0)**	1.2 (1.1–1.2)	0.9 (0.9–0.9)	—
1–11, 2022	**4.4 (3.8–5.2)**	4.2 (3.4–5.3)	4.7 (3.7–5.8)	—	**2.2 (2.0–2.3)**	2.7 (2.5–3.0)	1.6 (1.4–1.8)	—	**1.3 (1.2–1.3)**	1.4 (1.4–1.5)	1.1 (1.1–1.1)	—
12–23, 2022	**3.2 (2.8–3.7)**	2.9 (2.4–3.6)	3.5 (2.9–4.3)	—	**2.0 (1.9–2.1)**	2.6 (2.4–2.9)	1.4 (1.3–1.6)	—	**1.3 (1.3–1.3)**	1.5 (1.5–1.5)	1.1 (1.1–1.1)	—
24–36, 2022	**3.3 (2.9–3.7)**	3.4 (2.8–4.1)	3.2 (2.6–3.8)	—	**1.5 (1.4–1.6)**	1.9 (1.7–2.1)	1.1 (1.0–1.3)	—	**1.1 (1.1–1.1)**	1.2 (1.1–1.2)	1.0 (1.0–1.0)	—
37–53, 2022	**2.6 (2.3–3.0)**	2.8 (2.4–3.3)	2.5 (2.1–2.9)	—	**1.5 (1.5–1.6)**	1.9 (1.7–2.0)	1.2 (1.1–1.3)	—	**1.0 (1.0–1.0)**	1.2 (1.1–1.2)	0.9 (0.9–0.9)	—

**Abbreviations:** ED = emergency department; NSSP = National Syndromic Surveillance Program.

* Rate is the number of cannabis-involved ED visits per 10,000 ED visits. NSSP collects free-text reason for visit (chief complaint), discharge diagnosis, and patient demographic details. Free-text keywords and diagnostic codes combined using Boolean searches were used to create a keyword syndrome to identify ED visits involving cannabis. CDC developed and validated a definition for cannabis-involved ED visits using *International Classification of Diseases, Tenth Revision, Clinical Modification* diagnosis codes F12.1, F12.2, F12.9, or T40.7 or chief complaint text indicating cannabis use (e.g., “smoke weed” or “ingest hash”).

^†^ Visit ratios were calculated by dividing the rate of cannabis-related ED visits during the surveillance period by the rate of cannabis-related ED visits during the reference period. The reference period is the 2019 period (i.e., epidemiologic weeks) corresponding to the surveillance period. Ratios >1 indicate a higher rate of cannabis-related ED visits during the surveillance period than the reference period. Visit ratio analyses did not include weeks 1–11, 2020 as a reference period.

^§^ NSSP receives anonymized medical record information from approximately 75% of nonfederal EDs nationwide. To reduce the artifactual impact of changes in reporting patterns, analyses were restricted to facilities with more consistent reporting of more complete data (coefficient of variation ≤40 and average weekly informative discharge diagnosis ≥75% complete during 2019–2022).

^¶^ A standardized method of counting weeks to allow for the comparison of data year after year; epidemiologic weeks start on a Sunday and end on a Saturday. Four periods were analyzed: prepandemic (epidemiologic weeks 1–11), second half of school year (epidemiologic weeks 12–23), summer (epidemiologic weeks 24–36), and first half of school year (epidemiologic weeks 37–53).

** 95% CIs that exclude 1 are statistically significant.

^††^ Visit ratios by sex were calculated by dividing the rate of female cannabis-related ED visits during the surveillance period by the rate of male visits during the same period. Ratios >1 indicate a higher rate of female than male cannabis-involved ED visits during the specified surveillance period.

^§§^ Visit ratios were not calculated for weeks 1–11, 2020 because this surveillance period is still considered prepandemic.

Among children and adolescents aged 11–14 years, the pandemic peak in mean weekly visits (209.3) occurred during the second half of the 2021–22 school year (2022, weeks 12–23). Beginning in 2020, cannabis-involved ED visits also increased among persons aged 11–14 years, and during this time, visit ratios among females were higher (range = 1.5 [2020, weeks 37–53] to 2.7 [2022, weeks 1–11]) than they were among males (range = 0.9 [2021, weeks 24–36] to 1.6 [2022, weeks 1–11]). Within this age group, visit ratios by sex were not statistically significantly different during the early 2020 pandemic periods; however, beginning in the first half of the 2020–21 school year (2020, weeks 37–53), ED visit rates among females surpassed those among males and remained higher than rates among males throughout the study period.

More than 90% of cannabis-involved ED visits by persons aged <25 years occurred among those aged 15–24 years. The peak in mean weekly cannabis-involved ED visits among this age group (2,813.2) occurred during the second half of the 2020–21 school year (2021, weeks 12–23). Among children and adolescents aged 11–14 years, the peak (209.3) was approximately 7% of the peak among the older group and occurred 1 year later (2022, weeks 12–23). Rates of cannabis-involved ED visits were elevated among persons aged 15–24 years from 2020 through summer 2021 relative to reference periods (visit ratio range = 1.1 [2021, weeks 24–36] to 1.7 [2020, weeks 12–23]); however, rates briefly returned to baseline during the first half of the school year in both 2021 and 2022 (weeks 12–23) (Figure).

**FIGURE:**
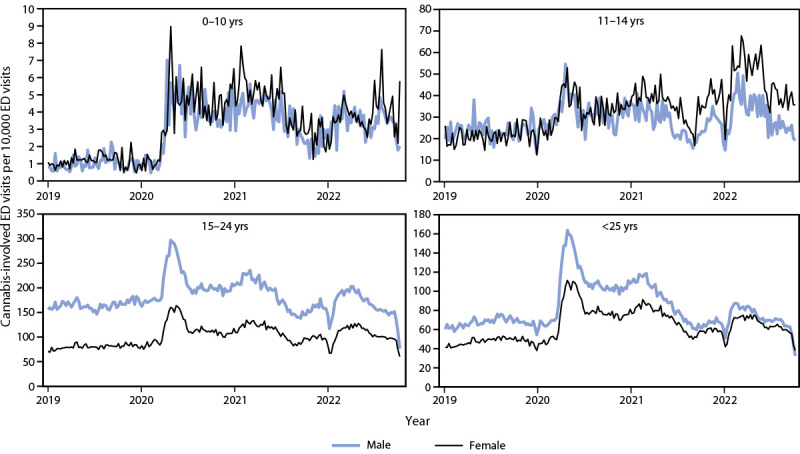
Weekly rates* of cannabis-involved^†^ emergency department visits^§^ among persons aged <25 years, by age group — National Syndromic Surveillance Program, United States, 2019–2022 **Abbreviations:** ED = emergency department; NSSP = National Syndromic Surveillance Program. * Number of cannabis-involved ED visits per 10,000 ED visits. ^†^ NSSP collects free-text reason for visit (chief complaint), discharge diagnosis, and patient demographic details. Free-text keywords and diagnostic codes combined using Boolean searches were used to create a keyword syndrome to identify ED visits involving cannabis. CDC developed and validated a definition for cannabis-involved ED visits using *International Classification of Diseases, Tenth Revision, Clinical Modification* diagnosis codes F12.1, F12.2, F12.9, or T40.7 or chief complaint text indicating cannabis use (e.g., “smoke weed” or “ingest hash”). ^§^ NSSP receives anonymized medical record information from approximately 75% of nonfederal EDs nationwide. To reduce the artifactual impact from changes in reporting patterns, analyses were restricted to facilities with more consistent reporting of more complete data (coefficient of variation ≤40 and average weekly informative discharge diagnosis ≥75% complete during 2019–2022).

## Discussion

Cannabis-involved ED visits began increasing statistically significantly among all age groups except 15–24 years several years before the pandemic (*3*), potentially as a result of expanding state-level policies legalizing cannabis use. Importantly, the current study found that cannabis-involved ED visits among all persons aged <25 years increased during the COVID-19 pandemic, and despite fluctuations, remained higher than 2019 prepandemic levels throughout 2022. The specific reasons for these increases are unknown, and potential drivers might differ by age.

Among persons aged ≤10 years, cannabis-involved ED visit rates during the pandemic far exceeded those preceding the pandemic; these findings are consistent with recent National Poison Data System data demonstrating that from 2017 to 2021, cases of edible cannabis ingestion among children aged <6 years increased by 1,375%, with statistically significant increases in toxicity and severity during the COVID-19 pandemic relative to those observed 2 years earlier (*4*). In June 2022, the Food and Drug Administration released a consumer alert^¶¶¶^ warning that THC-containing edibles are easily mistaken for products that might appeal to children and recommended that these products be kept in a safe place out of children’s reach, such as in a locked box. Strengthening policies requiring comprehensive labeling could also mitigate risk for unintentional ingestion.****

Cannabis-involved ED visit rates among children and adolescents aged 11–14 years also increased during the pandemic. Visit ratios by sex did not differ among children and adolescents aged 11–14 years until early into the pandemic; however, female cannabis-involved ED visit ratios surpassed those of males in the first half of the 2020–21 school year (2020, weeks 37–53), and this continued throughout most of the pandemic. This might indicate that females were more likely than males to use cannabis to cope with pandemic-related stress. Increased substance use by some young persons might be the result of pandemic-related stressors (*5*); a 2021 study found that during the pandemic, young females were more likely than males to use harmful coping mechanisms to address stressors and were more likely to require hospital admission for eating disorders (*6*). Implementation of evidence-based school-based programs designed to improve coping and problem-solving skills during adolescence have shown promise in preventing cannabis initiation and harmful use (*7*). Increasing substance use prevention efforts through youth-directed programming interventions might help address pandemic-related substance use.

Most cannabis-involved ED visits were among adolescents and young adults aged 15–24 years. More research is needed on age-related cannabis administration routes; however, administration routes that deliver higher concentrations of THC (e.g., vapes and dabs [highly concentrated extracts of THC derived from the marijuana plant]) are common among adolescents and young adults (*8*). Products with high THC concentration can increase the risk for excess consumption and lead to greater intoxicating effects (*9*). The largest visit ratios for this age group occurred immediately after the March 11, 2020, declaration of the pandemic as a public health emergency and during the initial implementation of many state-level stay-at-home orders (*10*). Monitoring the Future data on past-year marijuana use for 2020–2022 showed decreases in use by students in grades 10 and 12 during the pandemic, and slight increases in THC vaping by grade 10 students in 2022, although still below prepandemic levels. However, a National Institute on Drug Abuse analysis found that marijuana use among persons aged 19–30 years increased statistically significantly during 2021, reaching all-time high levels.^††††^ Thus, the observed increases among persons aged 15–24 years might be driven, at least in part, by use among persons beyond high school age.

### Limitations

The findings in this report are subject to at least four limitations. First, NSSP data are not nationally representative; results cannot be generalized to nonparticipating jurisdictions. Second, differential coding practices, changes in emergency care–seeking behavior during the pandemic, and fluctuations in the number of EDs participating in NSSP might underestimate or overestimate ED visits. However, analyses were restricted to facilities consistently reporting data during the study period. Third, although multiple visits by the same patient and the intent of cannabis use (i.e., intentional versus unintentional) cannot be distinguished, the data clearly illustrate patterns in cannabis-involved ED visits. Finally, syndromic surveillance data are updated in near real-time and are not considered final research data sets; results are likely to change as underlying medical record information is updated.

### Implications for Public Health Practice

Cannabis-involved ED visits among young persons increased during the COVID-19 pandemic and remained elevated above prepandemic levels. These increases might stem from multiple factors, such as increased use as a coping mechanism for pandemic-related stressors, use of highly concentrated THC products, increased availability of cannabis in states with legal marketplaces, and increased unintentional ingestions associated with packaging that is appealing or confusing to youths. To protect against unintentional ingestions of cannabis, it is important for adults who use cannabis to safely and securely store cannabis products in places inaccessible to children. Communities, schools, and coalitions (such as Drug-Free Community coalitions)^§§§§^ can implement evidence-based youth substance use prevention interventions to address changing patterns of cannabis use during the pandemic. These local organizations are best suited to meet youths in their communities and tailor interventions to effectively decrease cannabis use. States can implement or strengthen packaging restrictions to decrease youth appeal (e.g., plain packaging, comprehensive labeling, and more prominent warning labels). In combination, these strategies can help mitigate concerning rises in cannabis-involved ED visits among young persons.

## Footnotes

*
https://www.samhsa.gov/data/sites/default/files/2022-12/2021NSDUHFFRHighlights092722.pdf

†
https://www.ncsl.org/health/state-medical-marijuana-laws

§
https://monitoringthefuture.org/wp-content/uploads/2022/12/mtf2022.pdf

¶ To reduce artifactual impact from changes in reporting patterns, analyses were restricted to facilities with more consistent reporting of more complete data (coefficient of variation ≤0.40 and average weekly informative discharge diagnosis ≥75% complete during 2019–2022).

** NSSP is a collaboration among CDC, local, and state health departments, and federal, academic, and private sector partners. NSSP receives medical record data from approximately 75% of EDs across 50 U.S. states, the District of Columbia, and Guam, although fewer than 50% of facilities from California, Hawaii, Minnesota, and Oklahoma currently participate in NSSP. https://www.cdc.gov/nssp/index.html

††
https://www.cdc.gov/nssp/overview.html

§§ Rates were calculated by dividing the number of cannabis-related ED visits by the total number of ED visits for the relevant surveillance period among the population of interest (by age and sex).

¶¶ Visit ratios were calculated by dividing the rate of cannabis-related ED visits during the surveillance period by the rate of cannabis-related ED visits during the reference period. The reference period is the 2019 period (i.e., epidemiologic weeks) corresponding to the surveillance period. Ratios >1 indicate a higher rate of cannabis-related ED visits during the surveillance period than during the reference period. Visit ratio analyses did not include weeks 1–11, 2020 as a reference period.

*** Female-to-male visit ratios (the rate of female cannabis-related ED visits during the surveillance period divided by the rate of male visits during the same period) >1 indicate a higher rate of female than male cannabis-involved ED visits during the specified surveillance period.

††† A standardized method of counting weeks to allow for the comparison of data year after year; epidemiologic weeks start on a Sunday and end on a Saturday.

§§§ 45 C.F.R. part 46.102(l)(2), 21 C.F.R. part 56; 42 U.S.C. Sect. 241(d); 5 U.S.C. Sect. 552a; 44 U.S.C. Sect. 3501 et seq.

¶¶¶
https://www.fda.gov/food/alerts-advisories-safety-information/fda-warns-consumers-about-accidental-ingestion-children-food-products-containing-thc

****
https://emergency.cdc.gov/han/2021/han00451.asp

††††
https://nida.nih.gov/news-events/news-releases/2022/08/marijuana-and-hallucinogen-use-among-young-adults-reached-all-time-high-in-2021

§§§§
https://www.cdc.gov/drugoverdose/drug-free-communities/coalitions.html
